# Jailing is failing: psychiatry can help

**DOI:** 10.1192/bjb.2024.8

**Published:** 2024-10

**Authors:** Andrew Carroll, Adam Brett

**Affiliations:** 1 Swinburne University of Technology, Alphington, Australia; 2 Start Court, Perth, Australia

**Keywords:** Human rights, stigma and discrimination, rehabilitation, psychiatry and the law, mental health services

## Abstract

There is accumulating evidence that imprisonment is expensive but does little to address the underlying drivers of offending. At the same time, it is now recognised that a large proportion of prisoners are diagnosable with significant psychiatric disorders. In this piece we explore the potential role of psychiatry in addressing the societal challenge of a failing prison system. We argue that core psychiatric skills of engaging in balanced, values-based thinking and implementing sound clinical processes can play an important role in reducing reoffending risk. We briefly discuss some of the key challenges involved and outline several relevant service models.

The Justice Reform Initiative is a politically non-aligned advocacy group in Australia, comprising an alliance of professionals from diverse backgrounds with a shared commitment to effecting social change. It is driven by evidence that ‘Jailing is failing our nation on every front – it leads to more offenders committing more crimes, more disadvantage and more cost to the taxpayer’^1^ (p. 1). The Royal Australian and New Zealand College of Psychiatrists is one of its many supporter organisations – an acknowledgement that our profession has an important role to play in this area.

Certainly, the notion that jailing is indeed failing has likely occurred to any psychiatrist who has worked in prisons. In that context we encounter ‘revolving door’ patients: people challenged by the effects of mental illness, addiction and other complex needs that are inadequately met in the community, who return to custody with dispiriting regularity. We thus see the human costs of one of the defining ‘wicked problems’ of late capitalism.

## What can psychiatry offer?

What might it mean for jails to succeed rather than fail? How can the discipline of psychiatry contribute to such success? Philosophical thought and empirical research regarding supposed links between ‘deviance’ in the form of criminal behaviour on the one hand and mental disorder on the other has a long and often contentious history.^2,3^ An understandable temptation in the face of such complexity is to limit our ambit to the provision of treatment for mental illness for those who happen to be in custody and to leave the details of service systems and policy, as well as the challenge of reducing recidivism, to others. In our view this would be an abrogation of responsibility: to advocate properly for our patients we need to confidently assert that our skills and knowledge have applicability beyond the narrow scope of providing treatment for individual patients.

People living with the challenges – commonly in combination – of mental illness, addiction and neurodevelopmental disorders are significantly overrepresented in criminal justice systems.^4^ This fact alone places an onus on our profession to seek to improve such systems. Furthermore, we would argue that there are at least two core psychiatric skills that are especially relevant to the thorny challenges posed by our failing criminal justice systems. These skills are certainly not unique to our profession, but by dint of our training and experience tend to be well developed among psychiatrists.

The first is the skill of applying balanced thinking: adopting the flexibility of mind required to work with a diversity of values.^5^ It is in the nature of our craft that we are comfortable with grappling with competing interests, grey areas and continua rather than simple dichotomies. Such skills are crucial in the complex arena of criminal justice, particularly when mental health issues are in play. The systems in which justice-involved patients are embedded are tasked with managing competing imperatives: care and containment;^6^ risk management and recovery;^7^ punishment and rehabilitation. The effective navigation of such systems in a way that meets the ethical imperative to advocate for our patients requires a capacity for nuance, self-reflection and a comfort with paradox. A classic paper on the role of isolation (‘wet’) cells in the management of prison suicide^8^ showed the value of such balanced thinking thus:

‘There is something profoundly repugnant about having to place a suicidal prisoner in a wet cell, yet it is a practice which within the constraints of current prison policy is simply unavoidable on occasion if concerns for patient safety are paramount. To abstain from any willingness to apply such coercive means in order to preserve my own sense of being a benign figure free from any association with the oppressive nature of the prison regime would be to abandon the prisoner/patient to the possibility of further despair and hopelessness.’

Here, competing values of safety and autonomy are made overt, showing reflection on both the psychiatrist's own core ethical values and the broader issues (including policy constraints) at play. The capacity for such skilled values-based thinking is at the heart of good psychiatric practice – not just in ‘forensic’ spaces but more generally.

The second skill is the effective and pragmatic application of the clinical process itself, in contexts where a range of professionals from diverse agencies and with different skills, values and responsibilities are addressing a particular challenge. By ‘the clinical process’ we refer to the iterative, quality improvement cycle whereby a comprehensive needs assessment (based on clinical formulation and diagnosis) underpins a range of carefully coordinated interventions, the outcomes of which are systematically monitored before needs are assessed again. Whether the problem at hand is one of recovery from mental disorder in the narrow sense, or rehabilitation after criminal offending, a similar process is required. Such a process – simple in theory but invariably challenging in practice – can be especially difficult to implement when working with justice-involved patients. Except for that small subset of patients who are transferred away from prisons into long-term hospital care, the application of such a process in custodial settings tends to be stymied by:

- loss of continuity of care due to staffing changes and/or abrupt prison transfer decisions
- role confusion between different agencies (what is ‘offender rehabilitation’ work and what is ‘mental health treatment’?)
- constraints on information-sharing – even when patient consent is given
- competing demands, such as political pressures to maximise the time that offenders spend incarcerated versus the rehabilitative imperative to facilitate community transition by way of a period of supervised community living.

Both balanced thinking and the robust application of the clinical process can play a key part in the reform of our criminal justice systems, particularly as they affect people diagnosed with mental disorders. In outlining the relevance of psychiatric skills to the justice reform agenda, a useful organising principle is to consider their potential role in crime prevention. Although this clearly should never be the primary purpose of mental health services, the *realpolitik* is that sustainable political and public support for funding of services for justice-involved patients will be best secured by demonstrable positive outcomes for public safety.

## Primary prevention of offending behaviour

Primary crime prevention – stopping the problem before it happens – requires the addressing of factors that influence the long-term likelihood of future offending. The role of psychiatry in this space, as compared with social policy interventions, is clearly limited. Nonetheless, given the now strong evidence that adverse childhood experiences are powerful risk factors for risk of future offending^9^ it follows that investment in perinatal psychiatry and in services for children, adolescents and parents whose functioning is affected by mental disorder can make a significant contribution to crime reduction at a population level.

## Secondary prevention of offending behaviour

Secondary crime prevention addresses the needs of people identified to be at elevated risk of criminal offending. It is at this level that the role of psychiatry becomes more salient: it is also where ‘turf wars’ within the profession and ideological tussles become most apparent.

Although it remains contested in some quarters, the empirical evidence is now clear that, for a small but significant subset of persons with mental illness, there is an associated heightened risk of offending behaviour, in particular violence.^10^ Furthermore, evidence is emerging^11^ that clinical engagement of such patients is associated with reduced reoffending risk. The reasons for this reduction are likely to be complex but it is reasonable to assume that both amelioration of symptoms and provision of supports to address associated psychosocial impairments are involved.

It has long been noted, however, that this subset of high-risk patients faces what John Gunn, writing nearly 50 years ago, referred to as banishment pressure – the tendency of mental health services to withdraw care – which he viewed as ‘perhaps, the product of two forces: first stigma, but just as important, fear in the face of inadequate skills and resources’.^12^ Patients at risk of reoffending and of ‘banishment’ are usually not difficult to identify. It does not require specialist forensic assessors to determine that the common combination of persistent psychotic symptoms, addiction and personality dysfunction is grounds for concern.

When considering the skills and resources required to adequately support patients with such a set of needs, we must acknowledge that many, at least initially, will not readily adopt the role of a help-seeking ‘consumer’, spontaneously seeking assistance for their distress. Rather, they will often be in need of assertive, involuntary treatment under mental health legislation. Pressure against the use of involuntary treatment of mental illness, as part of such assertive care, is now stronger than at any time in our professional lives. Supported by policies and treaties at the highest level^13^ it is now routinely asserted by academics and policymakers that coercive care is ‘a failure of care’^14^ (p. 338). Such views are doubtless underpinned by entirely legitimate values, including concerns regarding the traumatic impacts of involuntary treatment on many patients. However, most psychiatrists continue to apply more balanced thinking to this issue, arguing that account must be taken of the full range of values and human rights involved;^15^ this includes the right of suffering patients to effective medical care, as well as the value of preserving public (and staff) safety. The ongoing development of multidisciplinary approaches to reduce coercive practices, to the fullest extent feasible, will of course remain a key task.^16^

The growing demonisation of coercive care is, we believe, directly relevant to the role of psychiatry in secondary prevention of offending. Systemic pressures against involuntary treatment may lead to that subset of patients who are at highest risk of offending being effectively disbarred from access to proper care: banishment pressure in action. Offending and incarceration are all too likely to follow. This possibility was anticipated some time ago:^17^

‘it is difficult to see how force can be eliminated completely from service provision when the well-being and safety of patients, carers and the general public is at stake. Paradoxically, the more health professionals withdraw from assertive and involuntary treatment in the name of recovery, the more likely that police and others operating outside the mental health system will be called on to assume a coercive role.’

We would agree: there is a real risk that well-intentioned efforts to eliminate all coercion from mental healthcare will inadvertently result in many of our most unwell patients being criminalised as police and prison officers take on the coercive role abrogated by clinical services. A key task for psychiatrists in the years ahead will be to use our skills in balanced, values-based thinking to advocate for the engagement of complex and vulnerable patients in clinical care.

Ideally, access to service provision for such patients would be determined by their clinical need, including their need for therapeutic security. Unfortunately, the relevant legislation in many jurisdictions does not allow for a flexible approach to placement: generally, legal factors (such as judicial determination of lack of criminal responsibility) determine which services are responsible, contrary to best practice models of care.^18^ Where possible, psychiatrists can advocate for service models based on clinical and security needs, rather than entirely predicated on legal categories. Even in such service models, however, treatment of most patients at elevated risk of offending will be delivered by general services rather than specialist forensic services. With appropriate support, most such patients can be effectively treated in general mental health services, rather than being siloed into the forensic sector. The challenges of stigma, fear and poor resourcing identified by Gunn, however, clearly remain. One way in which such challenges are being addressed in the Australian State of Victoria is through the creation of a small number of multidisciplinary forensic assessment and consultation teams led by consultant forensic psychiatrists but funded by and embedded within general mental health services. Although not yet formally evaluated, such services have been well-received and are going some way to the development of a more functional continuum of care for patients at elevated risk of criminal behaviour. The core psychiatric skill of modelling the appropriate balance between the values of risk reduction and patient autonomy have been critical to their functioning. In addition, having an embedded multidisciplinary team allows for more than merely the production of risk assessments: rather, there is a complete clinical process of assessment followed by the treatment of unmet needs (at least some of which will be dynamic risk factors for offending) and iterative reassessment.

## Tertiary prevention of offending behaviour

Tertiary prevention refers to interventions that aim to reduce the likelihood of reoffending in those who have already committed a crime.

In courts that deal with minor offending, two broad types of mental health service have developed: court liaison services and therapeutic courts. Court liaison services assess people in the courts with mental disorder and make recommendations for ongoing management; this can include diversion to hospital or community care. This type of work is usually acute with no ongoing follow-up. Such services have been shown to reduce the rates of imprisonment and appropriately divert offenders.^19^

Therapeutic mental health courts identify people with mental disorder and provide management plans that the court itself can monitor. Western Australia has a therapeutic mental health court that has been operating for over a decade and is the only one of its kind in Australia. The service model (the Mental Health Court Diversion and Support Program) is based on evidence from similar initiatives throughout the English-speaking world.^20^ Involvement is voluntary and individuals stay on the programme for about 6 months, prior to formal criminal sentencing; successful participation can improve sentencing outcomes, and hence reduce the likelihood of imprisonment. Central to the programme is a coordinated multidisciplinary, interagency management plan implemented in collaboration with the patient by a team led by a consultant forensic psychiatrist: the ‘clinical process’ in action. The programme seeks to address a broad range of unmet psychosocial needs, including direct provision of psychiatric care before linking in with general, community-based mental health services. It was initially a pilot programme but was made permanent after demonstrating that its participants showed a reduction in risk of offending and an improvement in mental health and quality of life; cost savings to the public purse have also been significant. This pragmatic attempt to ‘shut the revolving door’ has thus been shown to be good for patients and their families, and good for the community. At this level of more minor offending at least, the criminal justice system may allow rehabilitative values to largely override the considerations of retributive justice.

For offenders at the more serious end of the spectrum, where a period of incarceration is unavoidable, the value of a significant period out of custody but under close supervision as the final phase of a criminal sentence has long been recognised. However, over the past decade, political imperatives have made parole more difficult to achieve. Although the political logic for this has been a supposed emphasis on prioritising the value of public safety, legal experts have noted:

‘the purpose of parole is to promote public safety by supervising and supporting the release and integration of prisoners into the community, thereby minimising their risk of reoffending (in terms of both frequency and seriousness) while on parole and after sentence completion’ (ch. 1, para. 1.16).^21^

Thus, denying parole, or cutting it so short as to render it meaningless, may in the aggregate reduce public safety, notwithstanding the inevitable fact that a small number of offenders will reoffend while on parole. This conclusion is likely to be all the more relevant where high-risk offenders have an associated mental illness: the development of an effective therapeutic alliance with a community mental health service is much more likely to be achieved if established during parole, when supervision from correctional services can act alongside psychiatric services in a carefully boundaried fashion.

For high-risk offenders with enduring mental illness being supervised by community correctional services (whether by virtue of parole or other court mandate), an innovative programme – the Forensicare Serious Offender Consultation Service – has been developed in Victoria. This is led by a consultant forensic psychiatrist and works alongside community corrections (with full consent of the patient) to improve the delivery of mental health services. Among other things, it produces forensic mental health reports that assess clinical and psychosocial needs and facilitate referrals to general community mental health services. Numbers are small but the signs are that such brokerage has been valuable, tackling stigma and anxiety within both mental health and correctional services.

At a process level, for serious offenders with complex mental health needs, the core psychiatric skills of balancing competing values and implementing a clinical process are key to more effective tertiary prevention. If the primary social value of prisons continues to be seen as the provision of safe containment while a retributive sentence is served, opportunities for benefit (both economic and social) will continue to be missed; this is especially so for offenders with an enduring mental illness. While retributive values are prioritised, the systematic assessment of rehabilitative needs (including those that might drive reoffending) and attempts to address those needs will continue to be deferred until close to ‘earliest release’ date. This is inconsistent with any evidence-based approach to rehabilitation, potentially allowing criminogenic factors such as dysfunctional coping skills and poor capacity for self-management of mental illness to fester for years within the prison setting, and hence become more entrenched. The success of jurisdictions where imprisonment is seen as an opportunity for systematically planned rehabilitation from day one potentially points to a better way.^22^

## Conclusions

In 1968 the psychiatrist Karl Menninger wrote:

‘before we can diminish our sufferings from the ill controlled aggressive assaults of fellow citizens, we must renounce the philosophy of punishment, the obsolete, vengeful penal attitude. In its place we would seek a comprehensive, constructive social attitude – therapeutic in some instances, restraining in some instances, but preventive in its total social impact’^23^ (p. 280).

The ‘nothing works’ pessimism of the 1970s and ’80s,^24^ followed by neo-liberal policies with a misplaced faith in the supposed efficacy of punishment to reduce recidivism, led to this aspiration being marginalised. The Justice Reform Initiative embodies the return swing of the pendulum. Irrespective of an insatiable public appetite for retributive approaches to justice, the economic imperatives of our times mean that we can simply no longer afford to continue with grossly inefficient punishment-oriented approaches that are demonstrably ineffective.^25^

The challenges posed by criminal offending clearly amount to far more than a ‘health’ problem. Nonetheless, for a substantial subset of justice-involved people, the profession of psychiatry, applied through a public health/prevention lens, can be a key element of public policy. Given the simple fact that so many offenders have substantial mental health problems it cannot be otherwise. Most importantly, this is not a specialist ‘forensic’ task: the bulk of the work with mental health patients who offend or are at risk of doing so will continue to be done by general psychiatrists. We need to develop and evaluate a range of service models, based on sound clinical processes. We need to robustly promote a balanced approach to the ethical challenges posed by the vexed issue of involuntary treatment. Jailing is indeed failing: we must not fail to be part of the solution.

## Data Availability

Data availability is not applicable to this article as no new data were created or analysed in this study.

## About the authors

**Andrew Carroll** is an Adjunct Associate Professor in the Centre for Forensic Behavioural Science, Swinburne University of Technology, Alphington, Victoria, Australia. **Adam Brett** is a consultant psychiatrist in the Start Court, a magistrates court offering the Mental Health Court Diversion and Support Program for adult offenders in Perth, Western Australia.
